# Multi-Disease Data Management System Platform for Vector-Borne Diseases

**DOI:** 10.1371/journal.pntd.0001016

**Published:** 2011-03-29

**Authors:** Lars Eisen, Marlize Coleman, Saul Lozano-Fuentes, Nathan McEachen, Miguel Orlans, Michael Coleman

**Affiliations:** 1 Colorado State University, Fort Collins, Colorado, United States of America; 2 TerraFrame, Westminster, Colorado, United States of America; 3 Innovative Vector Control Consortium, Liverpool, United Kingdom; 4 Liverpool School of Tropical Medicine, Liverpool, United Kingdom; Centers for Disease Control and Prevention, United States of America

## Abstract

**Background:**

Emerging information technologies present new opportunities to reduce the burden of malaria, dengue and other infectious diseases. For example, use of a data management system software package can help disease control programs to better manage and analyze their data, and thus enhances their ability to carry out continuous surveillance, monitor interventions and evaluate control program performance.

**Methods and Findings:**

We describe a novel multi-disease data management system platform (hereinafter referred to as the system) with current capacity for dengue and malaria that supports data entry, storage and query. It also allows for production of maps and both standardized and customized reports. The system is comprised exclusively of software components that can be distributed without the user incurring licensing costs. It was designed to maximize the ability of the user to adapt the system to local conditions without involvement of software developers. Key points of system adaptability include 1) customizable functionality content by disease, 2) configurable roles and permissions, 3) customizable user interfaces and display labels and 4) configurable information trees including a geographical entity tree and a term tree. The system includes significant portions of functionality that is entirely or in large part re-used across diseases, which provides an economy of scope as new diseases downstream are added to the system at decreased cost.

**Conclusions:**

We have developed a system with great potential for aiding disease control programs in their task to reduce the burden of dengue and malaria, including the implementation of integrated vector management programs. Next steps include evaluations of operational implementations of the current system with capacity for dengue and malaria, and the inclusion in the system platform of other important vector-borne diseases.

## Author Summary

Emerging information technologies, such as data management system software packages, can help disease control programs to better manage and analyze their data, and thus make it easier to carry out continuous surveillance, monitor interventions and evaluate control program performance. This will lead to better informed decisions and actions. We have developed a multi-disease data management system platform with current capacity for dengue and malaria that supports data entry, storage and query. It also allows for production of maps and both standardized and customized reports. The system includes only software components that can be distributed without the user having to pay licensing costs. It was designed so that the user can adapt many aspects of the system to suit local conditions (for example roles and permissions, user interfaces and display labels and which functionality is included under a given disease) without having to involve software developers. In conclusion, we have developed a system capable of aiding disease control programs in their task to reduce the burden of dengue and malaria, including the implementation of integrated vector management programs. The next steps include operational implementations and evaluations of the current system with capacity for dengue and malaria, and the inclusion in the system platform of other important vector-borne diseases.

## Introduction

Emerging information technologies are improving our capacity to predict, prevent and control vector-borne and other infectious diseases [1]–[7]. In North America, for example, the United States Centers for Disease Control and Prevention has implemented a National Electronic Disease Surveillance System to promote rapid collection of standardized disease data [8]. Furthermore, the recent emergence of mosquito-borne West Nile virus in North America resulted in new electronic surveillance systems for mosquito-borne arboviruses both in the United States [9] and Canada [10]. This type of initiative is, however, most commonly achieved through systems that include software components with high acquisition and/or licensing costs, thus preventing system implementation in resource-constrained environments and limiting the potential for using the systems to address the problem of neglected tropical diseases. One way of overcoming this problem is to harness the explosion of new software products, for example those emerging from the open source community [11], that can be used and distributed without incurring licensing costs. Practical examples of this occurring in public health include the use of Google Earth to map dengue cases in Mexico and Nicaragua, examine the spatial spread of dengue in Mauritius and track polio cases in the Democratic Republic of Congo [12]–[15].

The Innovative Vector Control Consortium recognized the potential for using emerging information technologies to improve vector and disease control program performance and ultimately reduce the burden of tropical vector-borne diseases such as dengue and malaria [16]. This resulted in an initiative that led to the development of the software package described herein: a multi-disease data management system platform (hereinafter referred to as the system) with current capacity for dengue and malaria, and with potential for addition of other important tropical vector-borne diseases such as Chagas disease, human African trypanosomiasis, leishmaniasis, lymphatic filariasis and onchocerciasis. To some extent, the system builds upon previous experience with development and implementation of data management systems for malaria in southern Africa [4], [17]–[20]. Key project goals included 1) ensuring that the system can be distributed without the user incurring licensing costs, 2) producing a system that can be adapted to local circumstances by the user with no or minimal involvement of software developers, 3) achieving a user-friendly system to support data entry, storage and query, as well as production of maps and both standardized and customized reports and 4) delivering a system capable of enhancing the user's ability to carry out continuous surveillance, monitor interventions, evaluate control program performance and engage in evidence-based decision making.

## Methods

### System Development and Testing Strategy

System functionalities were developed in an iterative process over an 18-month period with close contact between software developers and subject matter experts including operational field input from Malawi, Mexico, Mozambique, South Africa and Zambia public health partners to ensure that development priorities were in line with actual needs. System functionalities were assessed by positive and negative testing conducted by an internal testing team. Downstream more extensive testing needs to include pilot implementations of the system in different operational settings with naïve users.

### System Architecture and Software Components

The system was developed with a 3-tiered architecture (data tier – application/business logic tier – presentation tier) and is comprised exclusively of software components that can be distributed to users without licensing costs. The data tier includes a PostgreSQL relational database [21] enhanced with the PostGIS extension [22] to support geographical data. The application/business logic tier includes Java [23] and Apache Tomcat [24]. The presentation tier uses Firefox [25] and is complemented by applications to support production of reports, BIRT [26], and maps, GeoServer [27] and OpenLayers [28]. To link the tiers, and to allow the user to make changes to the system that automatically are reflected across the 3-tiered architecture, the system includes TerraFrame's Runway SDK application [29]. Runway SDK also was used as a rapid development tool to facilitate the development process.

### System Requirements

The system requires a minimum of 2 GB RAM, 100 GB hard drive and Intel Core2 2.0 GHz to operate on a stand-alone machine (projected hardware cost of $500–600 per stand-alone desktop). It was developed for a Microsoft Windows XP (Microsoft Corporation, Redmond, WA) operating system but has been informally tested on and shown to function also for Windows Vista, Windows 7, Apple Mac OSX (Apple Inc., Cupertino, CA) and Ubuntu [30].

### System Installation and Licensing

The system installation package, which will be made available on DVD media, includes the system itself, the system manual and stand-alone versions of OpenOffice [31], to support export/import files if the computer is not already equipped with Microsoft Excel, the reporting tool BIRT, to allow the user to create customized report templates which then can be imported into the system, FWtools to assist the user in transforming spatial data into well known text (WKT) format [32] and the application QCal which is specifically designed to calculate dose/time response curves for insecticide resistance bioassays [33]. Downstream we plan to enhance the installation package by also including a stand-alone Dengue Models application, developed by the University of California at Davis and Infectious Disease Analysis, and consisting of upgraded versions of the CIMSiM and DENSiM simulation model applications [34]–[37].

Distribution and licensing of the system is currently executed through the Innovative Vector Control Consortium [38]. There is no cost associated with the license (the system is a royalty free, licensed software).

## Results

Here we describe key aspects of the developed multi-disease data management system platform with capacity for dengue and malaria. One important goal was to deliver a system that the user can, to the extent possible, adapt to local circumstances without involving software developers. Below, we highlight system points of adaptability and provide examples of system functionality that is re-used across diseases.

### Points of Adaptability in the System

#### Capacity for different implementation scenarios

The system can be implemented 1) on a single stand-alone computer, 2) in a client/server environment where the client (user) logs in remotely to the system or 3) as a pool of installations with one master and one or multiple dependent installations. For the third implementation scenario, the system includes extensive synchronization functionality.

#### Customizable system menus and functionalities by disease

**Figure 1:**
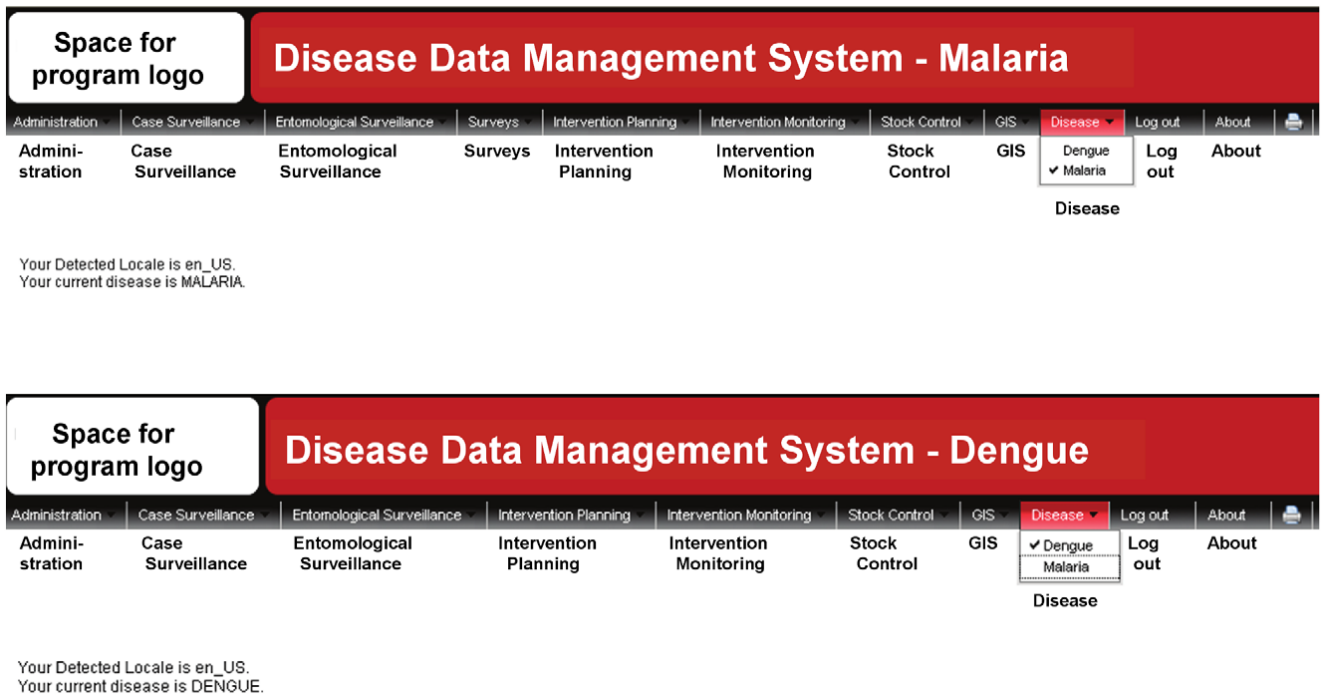
Main system menus for Malaria and Dengue.

One basic point of adaptability in the system is the disease of interest. This is handled through a menu item called Disease, where the user can select the disease of interest. Current options include dengue and malaria (**Figure 1**). Selection of a disease results in the user being presented with a menu that is configured specifically for that disease through the term tree (see section for term tree below). For example, the dengue menu for entomological surveillance includes a section for container collections of immatures (larvae and pupae of key dengue virus mosquito vectors are found primarily in water-filled artificial containers) which is not directly relevant for anopheline malaria vectors (where immatures typically are found in other types of water sources) and the section therefore is not included as a default option under the malaria menu. Conversely, the malaria menu includes a section for intervention monitoring related to use of intermittent preventive treatment (with anti-malarial drugs) of pregnant women, which is not relevant for dengue because drug treatment is still unavailable for this disease. However, if these situations were to change in the future, the user then can choose to include container collections of immatures in the malaria menu or intervention monitoring related to intermittent preventive treatment in the dengue menu. This, together with the capability outlined below to specify different label names for a data entry field by disease, provides system flexibility in that the user can adapt the default system to make use of functionality originally developed for one disease in another disease. It also provides an economy of scope as new diseases downstream can be added to the system at decreased cost through re-use of already existing functionalities.

#### Customizable user interfaces by disease

**Figure 2:**
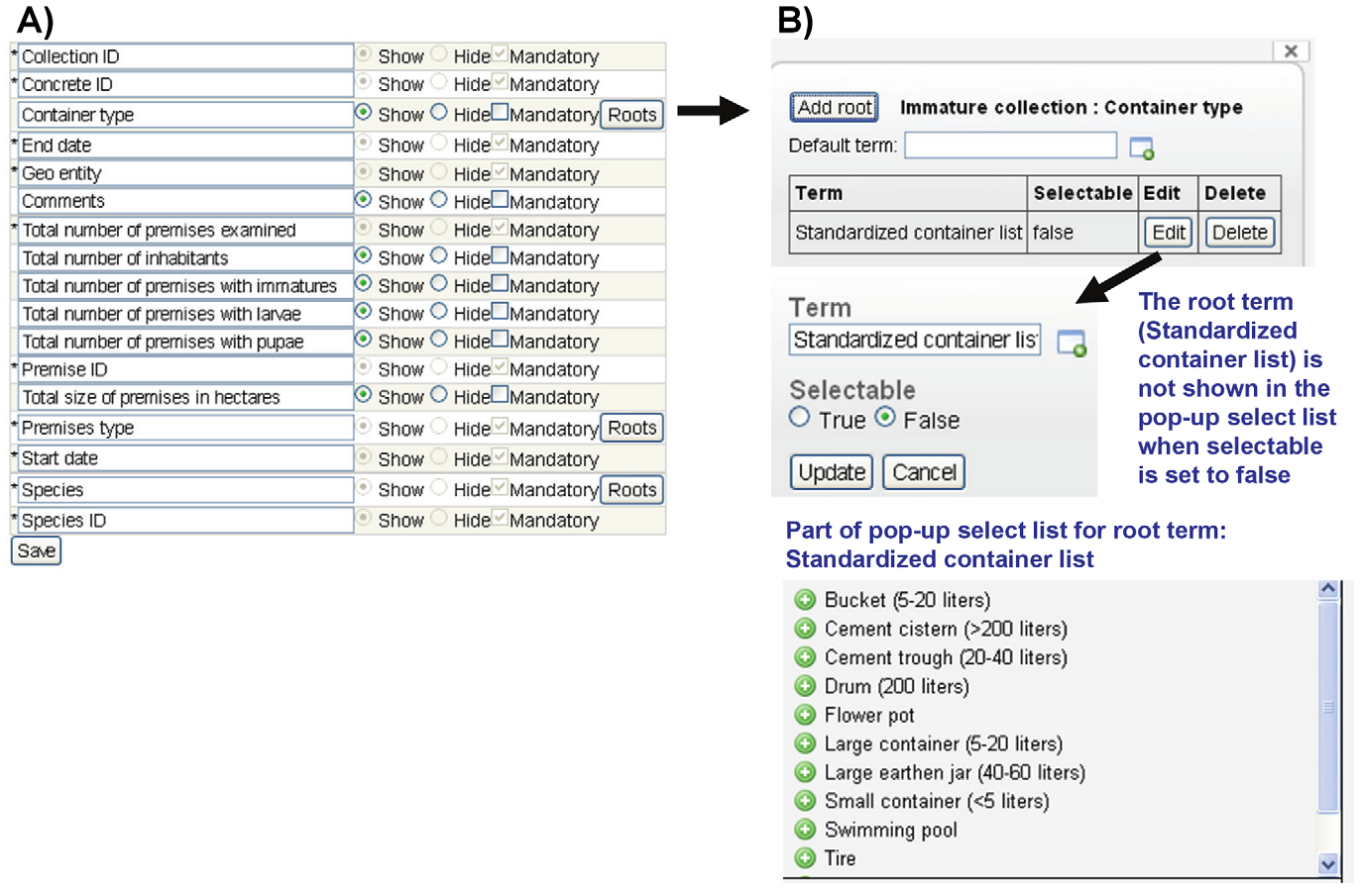
Example of functionality to customize the user interface and set term tree roots for pop-up select lists. A) Assign user interface visibility for mosquito immatures for data aggregated to container type. B) Add or edit root from term tree for pop-up select list.

Another important point of system adaptability is the capacity to customize user interfaces. The user can select, by disease, whether to show or hide a given data entry field (**Figure 2A**). This provides the user the option to hide default data entry fields that are not considered relevant for a given disease or in a given geographical area where the system is implemented, and thus produce a streamlined user interface. In the same functionality, the user also can set the term tree root (see section for term tree below) used for a given data entry field (**Figure 2B**).

#### Customizable system display labels by disease and language

The current system includes dengue and malaria and the default language is English. When applicable, a data entry field may have different display label names in the dengue and malaria menus. For example, for information regarding an individual disease case the default display label for causative agent is dengue virus serotype in the dengue menu but malaria type in the malaria menu. The user can change these disease-specific display labels in a localization spreadsheet that can be exported, edited and then imported back into the system to execute the changes. This localization spreadsheet can also be used to develop display labels, by disease, for another language, including language dialects (i.e., languages or dialects based on the Latin character set). The user then can switch between languages to be displayed by changing the browser locale in the Firefox browser.

#### Customizable system roles and permissions

**Figure 3:**
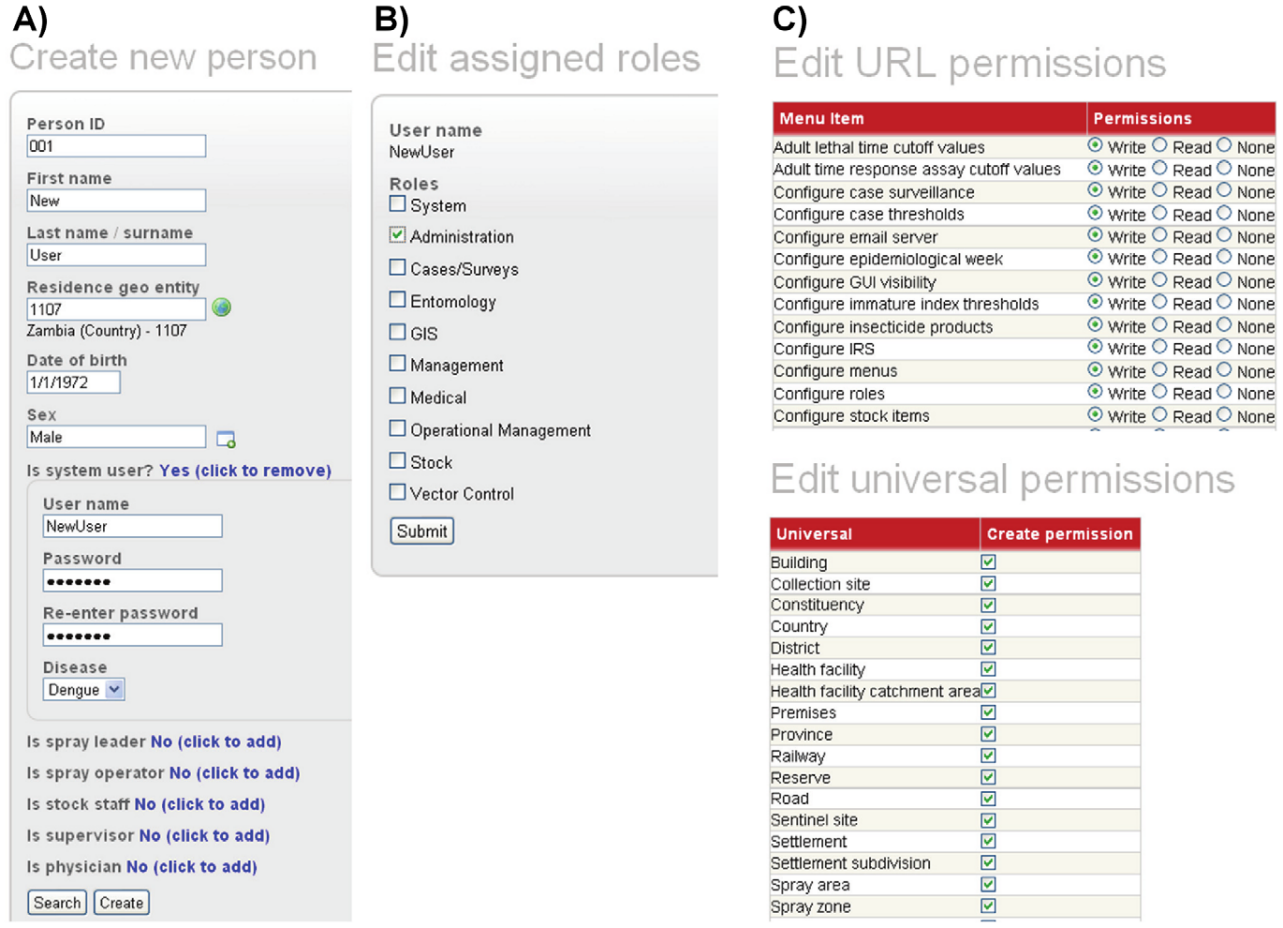
Administration functionality to customize system roles and permissions. A) Create new user. B) Assign role(s) for new user. C) Edit permissions for Administration role.

Another point of adaptability in the system is the administration functionality for customization of system roles and permissions (**Figure 3**). The system is delivered with a set of default roles (administration, entomology, medical, vector control, stock, etc.) but these are completely configurable in that the user can 1) change the names of existing roles, 2) create new roles and 3) define separate permissions for each role to a) access or work with different parts of the system (Write, Read, None/No access) and b) create entities against system universals (see section for universal tree below) (**Figure 3C**). This allows a system administrator to define a set of locally relevant roles that, for example, can be used to restrict access to sensitive information such as patient data. The system administrator can further refine the permissions by generating individual log-in names and passwords for each person using the system (**Figure 3A**). This allows for assigning individual system users a single role (e.g., administration or medical) or a combination of multiple roles (e.g., entomology, vector control and stock) (**Figure 3B**).

#### Customizable information trees – geographical entity tree

The geographical entity tree, which provides a spatial representation of the area in which the system is implemented, is entirely user configurable. The system default is a single root entity called Earth under which the user can build a locally relevant geographical entity tree, based on a located_in relationship structure, either manually or through successive imports of geographical data at increasingly fine spatial resolutions. This could, for example, start with geo entities representing countries followed by states, counties, settlements (cities, towns or villages), settlement subdivisions, and, if geographical data are available and relevant at such fine resolutions, geo entities representing blocks and individual premises (households). Each entity in the geographical entity tree is an instance of a universal term (e.g., the geo entity United States of America is an instance of the universal term Country).

**Figure 4:**
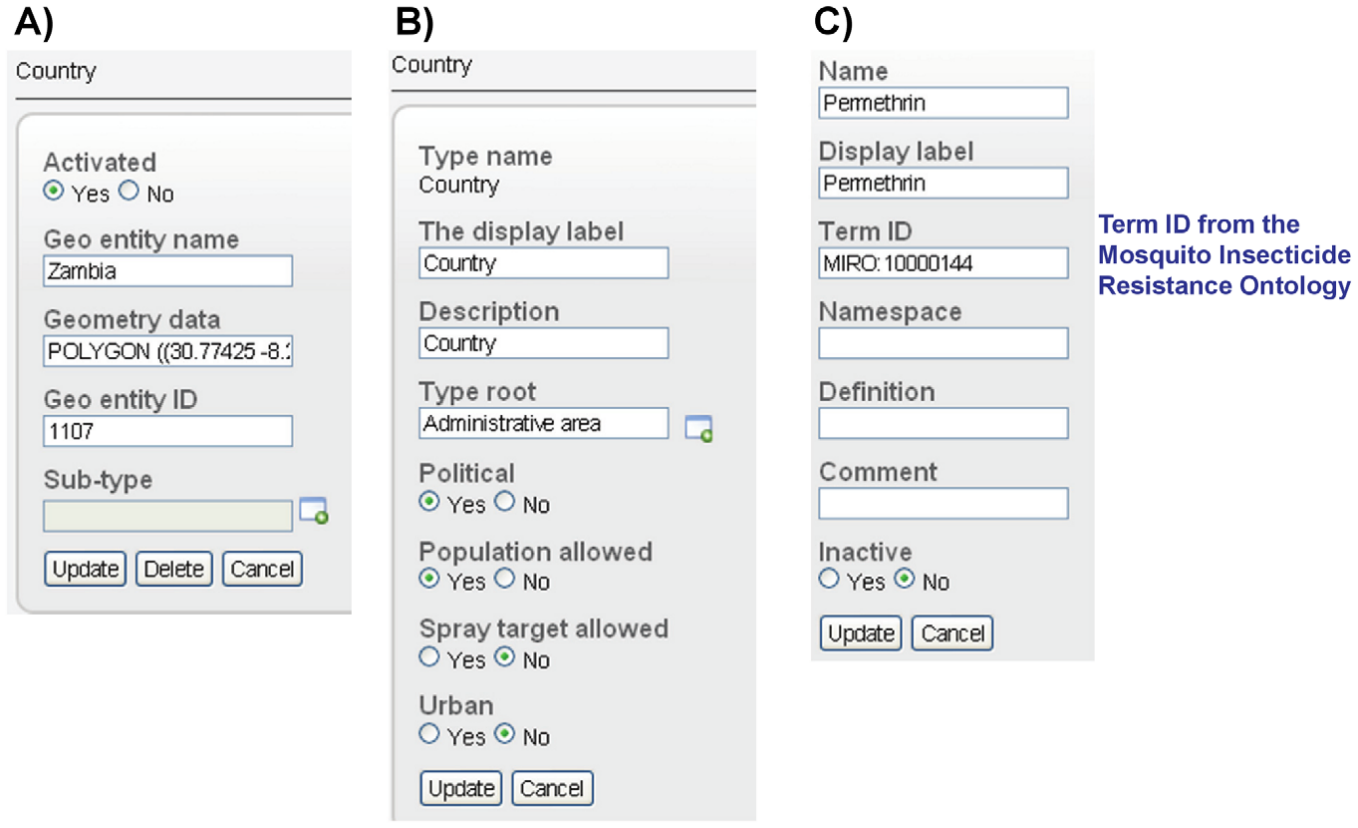
Editing options for geo entities, universals, and terms from the term tree. A) Editing options for geo entities. B) Editing options for universals. C) Editing options for term tree terms.

Import of data for geo entities, which typically will be derived from an external Geographic Information System (GIS) database, can include universal type, geo entity status (active/inactive), name and ID, type of geometry (point, polygon, etc.) and spatial data (in WKT format). After the geographical entity tree has been configured, the user can manually 1) add new geo entities, 2) edit the information for existing geo entities (**Figure 4A**) and 3) delete existing geo entities. One of the benefits of the geographical entity tree, derived from its located_in relationship structure (e.g., Colorado located_in United States of America), is the potential for aggregating data to coarser spatial resolutions in the system's internal data querying tools. For example, data entered against geo entities representing settlements in the data entry screen can be shown for settlement in the data querying tool but also can be aggregated to any coarser spatial scale such as county, state or country in the geographical hierarchy outlined above.

#### Customizable information trees – universal tree

As used in the system, universals are key spatial concepts that can be defined with regards to how they are used to support system functionalities. Six universals are required in the system to support specific functionalities and therefore cannot be deleted from the universal tree. These are health facility, collection site, sentinel site, spray zone, stock depot and surface (surface is used in insecticide efficacy bioassays). These typically will be complemented in the system by a set of user-created universals to mirror the structure of the locally relevant geographical entity tree, for example country, state, county, settlement, settlement subdivision, block and premises. Each of these universals can then be defined with regards to 1) if population is allowed, which is a requirement for calculation of disease case incidence against the universal, 2) if spray targets are allowed, which is a requirement for planning and monitoring of indoor residual spraying against the universal and 3) whether they are considered part of one or both of two geographical hierarchies that support different system functionalities: a) the political hierarchy and b) the urban hierarchy (**Figure 4B**).

#### Customizable information trees – term tree

**Figure 5:**
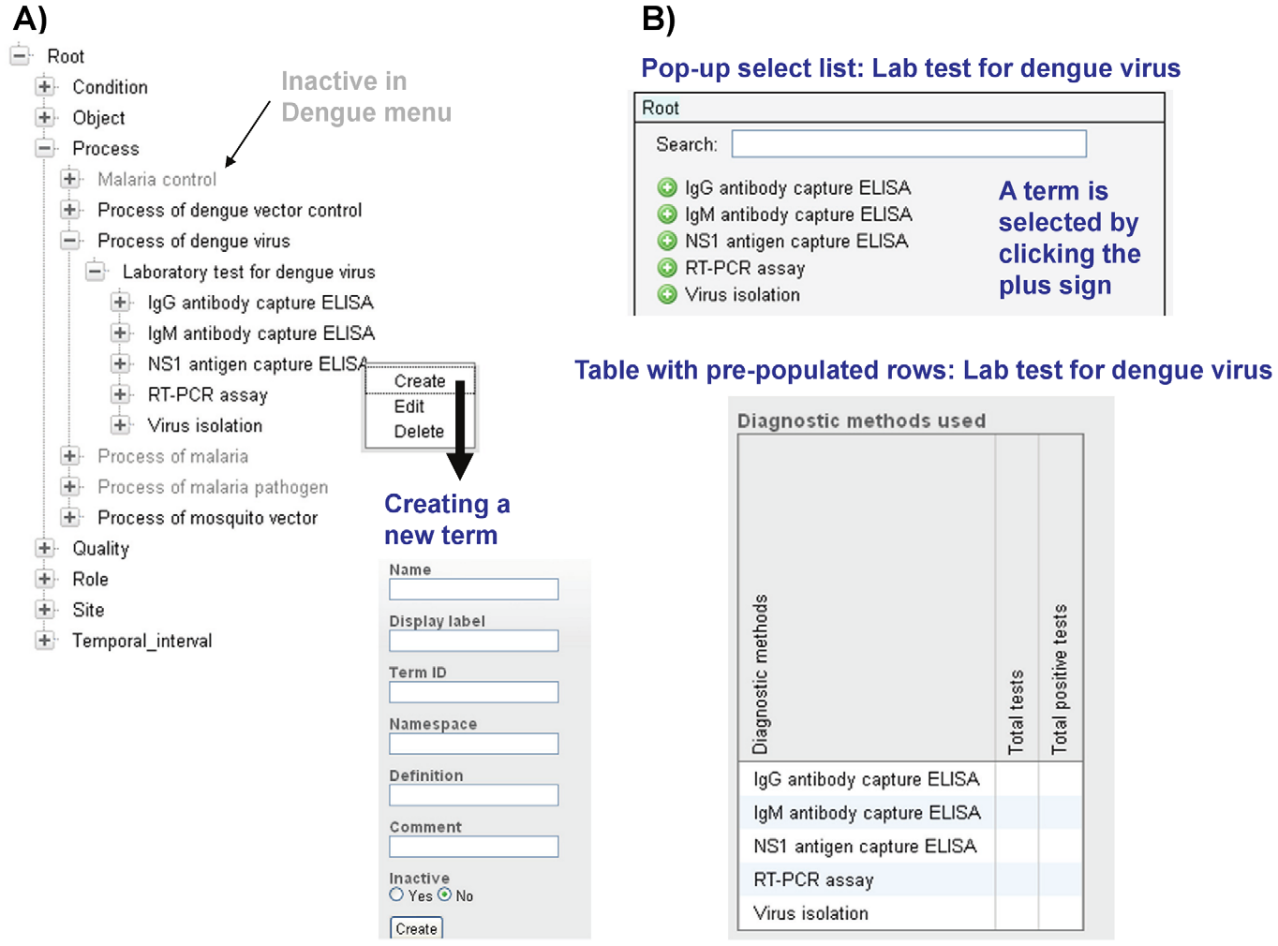
Example of term tree structure for Dengue and use of configurable term tree lists in data entry screens. A) Example of term tree structure. Terms that are active for Dengue are shown in black, those that are inactive for Dengue are shown in gray. B) Using the term tree in data entry screens.

One of the most powerful points of adaptation in the system is the user-configurable controlled vocabulary term tree. The term tree is based on ontological principles, following the Open Biomedical Ontologies [39], and is used in the system to define 1) options in pop-up select lists for data entry fields and 2) pre-configured entries for rows and/or columns in data entry tables (**Figure 5B**). An ontology is a set of standardized and logically defined terms (controlled vocabulary) and their inter-relationships. The controlled vocabulary term tree is built on a single ontological relationship, is_a; for example *Anopheles gambiae* is_a *Anopheles gambiae* sensu lato (the mosquito species *Anopheles gambiae* is a species in the *Anopheles gambiae* sensu lato complex). The system is delivered with a default term tree and each data entry field or row/column configuration in a data entry table that is populated from the term tree has a pre-configured root term that defines what is included in the select list for the data entry field or which terms that are used to define table rows/columns. Pop-up select lists may include terms found under one or multiple root terms; in the latter case the root terms themselves typically are included as the first visible level in the pop-up select list. Importantly, both the term tree itself and the selection of root terms are completely configurable by the user (**Figures 2B** **,** **5A**). This includes the ability to make terms active or inactive by disease (**Figure 5A**).

The term tree has multiple benefits. First, the terms included in the term tree represent standardized data across a system implementation. Each term is given a name, display label, and ID, and the user also can provide a definition for the term (**Figure 4C**). Use of standardized and well defined terms provides potential for sharing of data with related database initiatives. For example, we are making extensive use in the term tree of terms related to insecticide resistance that were derived from the Mosquito Insecticide Resistance Ontology (**Figure 4C**) and are used in the IRbase global database for insecticide resistance in mosquito vectors [40]. This will facilitate the process of bi-directional data sharing between IRbase and our system. Terms are also drawn from the emerging malaria ontology IDOMAL [41] and an ontology for vector surveillance and management under development at Colorado State University.

Second, the term tree moves the system away from hard-coded select lists and towards user-configurable select lists which facilitates the process of adapting the system to local conditions. It also provides an opportunity to design term tree entries that resolve practical problems such as overlapping definitions. Consider the example of the World Health Organization (WHO) definition for dengue disease manifestations which since 1997 has been dengue fever – dengue hemorrhagic fever [42] but now is proposed to change to dengue – severe dengue [43]. The term tree provides an opportunity to develop a branch called dengue disease classifications under which root terms can be added for the 1997 WHO classification system (including the terms: dengue fever and dengue hemorrhagic fever), 2009 WHO classification system (dengue and severe dengue) and a combined 1997/2009 WHO classification system with four possible combinations (dengue fever/dengue, dengue fever/severe dengue, dengue hemorrhagic fever/dengue, and dengue hemorrhagic fever/severe dengue). Information combining the 1997 and 2009 classifications, which as shown above can be handled through the term tree, may become important for global reporting purposes in a transition phase where different classification systems are used by different countries.

Third, the terms are used to pre-populate row and/or column fields in data entry tables (**Figure 5B**). This allows the user to dynamically change both the number of rows/columns that appear in a table and their respective header labels simply by making changes to the term tree content under the root term that is used to populate that specific table. The most powerful example comes from data entry for aggregated disease cases which includes multiple tables where both the rows and the columns are dynamically populated from the term tree. For example, the table for data by disease manifestation has rows that can be configured through the term tree, e.g., set to dengue fever and dengue hemorrhagic fever if the 1997 WHO classification system is followed, as well as columns that can be configured through the term tree to include patient categories of interest such as confirmed cases, clinically diagnosed cases, confirmed cases with dengue virus 1, 2, 3, or 4, etc. This type of dynamic table provides exceptional potential for system adaptation to local conditions without the involvement of software developers.

Fourth, the term tree is constructed based on the is_a ontological relationship which enables aggregation to higher levels in the term tree. Perhaps the best example here is the section of the term tree that deals with mosquito taxa. For example, based on the term tree is_a relationship, the system recognizes the malaria vector *Anopheles gambiae* as a species within the *Anopheles gambiae* sensu lato species complex (*Anopheles gambiae* is_a *Anopheles gambiae* sensu lato). It also recognizes *Anopheles gambiae* chromosomal forms and molecular forms as subtypes of *Anopheles gambiae*. This enables the system to 1) display records entered against either *Anopheles gambiae* sensu lato or any species/species subtype within this complex, such as *Anopheles gambiae* and its chromosomal or molecular forms, when a data record search is conducted for *Anopheles gambiae* sensu lato and 2) include data entered against any species/species subtype within the *Anopheles gambiae* sensu lato species complex when abundance calculations in the querying tool are defined to be executed at the taxonomic level of *Anopheles gambiae* sensu lato. Stock management is another example of system functionality that logically will include hierarchical levels for stock items defined in the term tree (for example equipment – mobile communications equipment – cell phones) and therefore benefits greatly from the use of the is_a relationship in the term tree.

#### Customizable pathogen transmission season and epidemiological week

Transmission seasons may vary for different vector-borne pathogens in a given geographical area, or for a given pathogen in different geographical areas. The system therefore allows the user to define a set of locally relevant transmission seasons, by disease, which then are used for planning of vector control interventions and to set alert thresholds for disease cases within a transmission season. A transmission season can fall within a calendar year or span 2 calendar years. Furthermore, epidemiological week can be configured by the user based on local conventions for which day of the week is considered the starting point for the epidemiological week.

#### Customizable alerts

Automated alerts that are triggered when system thresholds are reached or exceeded is perhaps the clearest example of decision support in the system. Such alerts are currently included for 1) disease cases and 2) abundance indices for container-inhabiting mosquito immatures. In both cases the thresholds can be configured by the user to suit local conditions so that alerts are not excessive to the point of being meaningless due to lack of resources to respond to them. The alerts for disease cases are triggered in real time, in the sense that the alert is triggered based on entry of the individual case that causes the threshold to be reached. If disease cases are entered into the system on a timely basis, these alerts can be used to facilitate outbreak response.

#### Adaptability of disease case alert thresholds

Alert thresholds are set by disease and geo entity or health facility, and the process of generating these thresholds has multiple points of user adaptability. First, the thresholds can, based on the user's preference, be calculated using different algorithms including mean +1.5 SD, mean +2 SD, modified binomial 95%, modified binomial 99% and the upper third quartile [17]. Two of these options can be included as separate threshold alert levels called 1 and 2. Second, based on what type of historical data have been entered into the system, the user can select to calculate the thresholds based on data for aggregated cases, individual cases, or individual and aggregated cases combined. The user also can select to calculate thresholds against presumed source of infection (a geo entity representing an administrative boundary unit) and/or health facility (where the case was reported). Third, the user can both define the number of previous transmission seasons for which to include data for the threshold calculation and provide a weight for each transmission season to address the effect of outliers with unusually low or high case loads. Fourth, the user can define the number of weeks preceding and following the epidemiological week that is included in the threshold calculations; this is particularly useful for scenarios where the epidemiological/epidemic curve may temporally shift slightly between transmission seasons, for example due to variation in when the rainy period starts and vector abundance and pathogen transmission intensity begins to increase. Fifth, the system calculates which percentage of clinically diagnosed cases that should be included in the threshold calculation based on historical data in the system for confirmed positive versus conformed negative cases. This is particularly useful for arboviral diseases, such as dengue, where disease outcomes range from mild to severe manifestations, the initial clinical diagnosis often may prove incorrect and many clinically diagnosed patients do not return to provide convalescent samples for confirmatory tests of virus exposure. Sixth, the system provides the user the additional option to manually enter or edit threshold values, by geo entity (source of infection or health facility), transmission season, and epidemiological week for thresholds alert levels 1 and 2.

#### Adaptability of triggering of disease case alerts

The system keeps track of a current case count, by disease, which is updated every time a new individual case is entered into the system and an alert is triggered on the case entry for which the threshold is reached for a given disease in a given geo entity or health facility. The user can adapt several aspects of how disease case alerts are triggered. First, the user can select whether to base the current case count on fixed epidemiological weeks or sliding weeks (i.e., including cases occurring from six days prior to the date of onset of symptoms for a given case). Second, the user can define which percentage of clinically diagnosed cases that should be used for calculation of the current case count. This is especially useful for diseases where laboratory confirmation often takes one or several weeks and where only partial data, including a clinical diagnosis, initially is entered for a case which then later can be updated with information on a positive or negative confirmed diagnosis. Because the percentage is set manually, the user also can change it temporarily in response to extraordinary events such as outbreaks of other diseases resulting in increased levels of clinical misdiagnosis. Third, alerts can be activated in the system for source of infection and/or health facility.

### Data Entry, Data Querying, Pre-Defined Custom Calculations and Reporting/Mapping

To minimize data entry error, data entry fields make extensive use of geo entities selected from the geographical entity tree, dates selected from pop-up calendars and pop-up select lists from the term tree. To facilitate use of the geographical entity tree and term tree, which can be tedious to navigate in order to find a specific geo entity or term, the user can simply start typing the name of the geo entity or term that a field should be populated with and be presented with a drop-down list containing all possible options based on what has been typed in so far. The desired geo entity or term can then be selected from the drop-down list.

**Figure 6:**
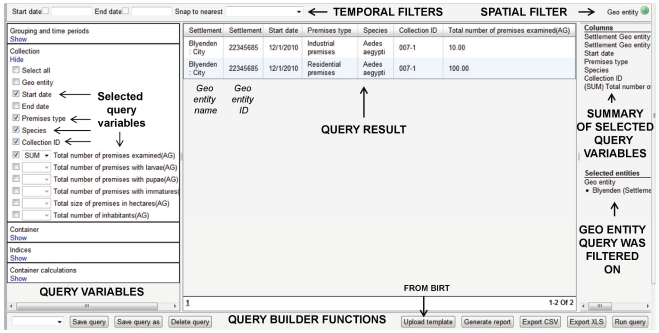
Example of query builder screen. Notations indicate functions in different parts of the query builder screen.

Data querying is accomplished through a set of unique system tools referred to as query builders where the user can define a specific data query (**Figure 6**). Separate query builders were developed to handle different functionalities, for example individual disease cases, aggregated disease cases, insecticide resistance bioassays, intervention monitoring relating to indoor residual spraying, etc. All query builders include the capacity to filter a query on 1) start and end dates, 2) geo entities from the geographical entity tree, 3) terms from the term tree, corresponding to the term options available for the data entry field, for query fields that relate to the term tree, 4) specific values included in hard-coded select lists and 5) numeric values or ranges for query fields based on numerical data (**Figure 6**). This provides exceptional potential for the user to easily and rapidly define and execute specific data queries.

**Figure 7:**
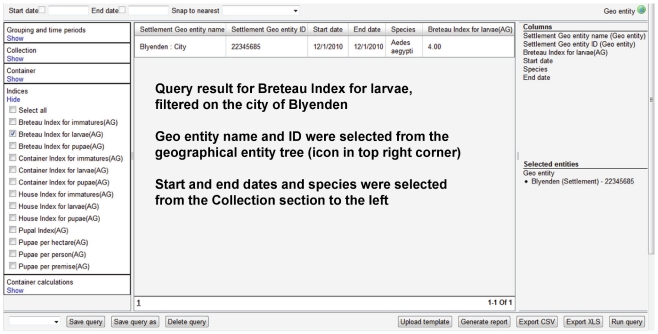
Example of pre-defined custom calculations in a query builder. Query result for Breateau Index for larvae.

Many of the query builders also include the option of executing pre-defined custom calculations. For example, the query builders for disease cases include custom calculations for case incidence and case fatality rate, and the query builder that handles information for container collections of mosquito immatures includes custom calculations for commonly used indices such as Breteau index, container index, house index, and pupae per person (**Figure 7**). These calculations also can be aggregated to coarser geographical scales than the one at which the data were entered. For example, data entered against county would allow for calculations to be executed against county, state, or country.

The functionality relating to pupae recorded by individual container provides a powerful example of the dynamic linkage between the term tree, the data entry screens and the query builders. The data entry screen includes a table where each row represents an individual container and where the numbers of pupae collected by mosquito taxon are entered in columns that are created dynamically from a list of taxa defined under a term tree root (default list is *Ae. aegypti*, *Ae. albopictus*, and *Aedes* spp.). The corresponding query builder includes a separate section for each of these taxa with options for summary numerical data and a set of pre-defined custom calculations. The inclusion of Runway SDK to drive system operations allows for an automatic, adaptive process where the user only needs to go into the term tree and inactivate a default taxon or add a new taxon in order for the system to automatically 1) update the dynamic columns in the table in the data entry screen and 2) add or remove the section for the affected taxon from the query builder.

All query builders also include options to 1) export query results as .csv or .xls files, 2) save and re-use specific querying field combinations that are executed on a regular basis and 3) upload pre-configured BIRT report templates and use these to produce standardized reports (see buttons at the bottom of the query builder shown in **Figure 6**). Mapping is directly linked to the query builders in that the map generation process makes use of information that is saved in the query builders as specific named query results. Importantly, mapping supports multiple layer views from different query results that may have been produced through a single query builder or several different ones, for example combining results for entomological surveillance, disease case surveillance and intervention monitoring in a single map. The system also supports export of spatial data in commonly used formats such as shapefile or Keyhole Markup Language (KML) file.

### Re-Used System Functionalities Across Diseases

The multi-disease platform includes functionalities that are entirely or in large part re-used across diseases. This includes the administration functionalities, stock management, GIS, case surveillance (re-used in large part across dengue and malaria), and most of the functionalities relating to entomological surveillance. Because the platform at this point only includes two diseases, some of the functionalities are relevant only for dengue or malaria. However, as more vector-borne diseases are added to the platform, these functionalities also will become re-used across diseases. For example, container surveillance of mosquito immatures would be directly applicable to other arboviral diseases where the causative agent is transmitted by *Ae. aegypti*, such as chikungunya or yellow fever. Intervention monitoring provides other examples of functionalities that will be re-used when additional vector-borne diseases where similar control methods are employed are added to the system platform.

Some data in the system are shared between diseases because they are relevant across diseases. This includes universal terms and human population data recorded against geo entities representing administrative boundary units or health facilities. Other data are shared across diseases but can be made active or inactive by disease; this includes, for example, the terms in the term tree.

### Examples of Disease-Specific Functionalities in the Default System for Dengue and Malaria

To avoid cluttering the menu for a given disease with functionalities that are unlikely to be relevant for that disease, the system comes with a default set of functionalities by disease. Functionalities which in the default system for dengue and malaria are included only for the malaria menu include 1) surveys, where the data captured largely conform to the malaria indicator survey developed by the Roll Back Malaria Partnership's Monitoring and Evaluation Reference Group [44]–[45], 2) planning and monitoring of indoor residual spraying programs, 3) monitoring of interventions based on use of insecticide-treated nets or intermittent preventive treatment for pregnant women with anti-malarial drugs and 4) monitoring of control of anopheline mosquito immatures that inhabit non-container aquatic habitats, which may range in size from cattle hoof prints or small puddles to rice fields and lake shores. Functionalities which are included only for the dengue menu include 1) container-based surveillance of immatures of key dengue virus vectors, such as *Ae. aegypti* and *Ae. albopictu*s, which exploit a wide range of containers (e.g., water storage containers, tires, bottles, cans, flower pots, etc.) as larval development sites [46]–[47] and 2) capture of summary data, by individual premises or aggregated to larger spatial units such as blocks or neighborhoods, for intervention methods used in a given geographical area during a specific time period.

### Potential for Use in Integrated Vector Management

Our multi-disease system incorporates a broad range of information including entomological, epidemiological, stock and spatial data. This sets the stage for using the system to support integrated vector management (IVM) which uses a wide range of interventions, often in combination and synergistically [48]–[49]. For example, the functionality for intervention monitoring in the dengue menu allows for capture of data, in space and time, for different implemented intervention methods (the methods are defined by the user through the term tree) used as parts of an IVM program. These data, together with data for entomological and epidemiological surveillance captured in other parts of the system, can then be used to assess the impact of the IVM program on entomological and epidemiological outcome measures such as vector abundance or presence or prevalence of infection in the human population.

## Discussion

We report on the successful development of a multi-disease data management system that can be distributed without the user incurring licensing costs, and that has exceptional potential for adaptation by the user to local circumstances, allowing resource poor countries to benefit with minimal expense. The system incorporates user-friendly functionalities for data entry, data storage, data query, mapping and reporting. We consider it likely that use of the system will lead to improved continuous surveillance, intervention monitoring and evaluation of control program performance. However, this needs to be confirmed through operational implementations of the system.

As the Results section already extensively highlights the strengths of the system, the Discussion section focuses more on system limitations and potential for improvement.

### Operational Testing of the System

Plans are now underway to trial the system operationally with control program partners so that it can be rigorously evaluated. Ideally, the system should be evaluated in parallel with existing management systems for a 1–2 year period in multiple settings for each relevant disease. This will also provide valuable feedback on system performance and lead to improvements in later versions of the system.

### System Complexity and Adaptability

The system has exceptional potential for adaptation by the user to local circumstances without the involvement of software developers. The cost of this versatility is increased complexity for the system administrator. Implementation and operational use of the system without a highly competent local system administrator is likely to result in poor system performance, especially with regards to synchronization of data. The complexity is most apparent during the initial system configuration.

### Data Import

The system includes extensive capacity for data import, including import spreadsheets that are tailored to specific functional components such as entry of individual disease cases, entry of data for insecticide resistance bioassays, entry of survey data, entry of data for distribution of insecticide-treated nets, etc. This allows the user to rapidly populate the system with historical data. However, it should be noted that the import process, for data quality purposes, is unforgiving when it comes to poor quality data. Therefore, it often will be necessary to clean historical data that originate from other sources, which may not enforce input of high quality data, before importing the data into the system. Furthermore, the behavior of the import process for a given import spreadsheet is linked to the behavior of the corresponding data entry screen, for example with regards to data fields where an entry is mandatory. Thus, it becomes important that all personnel executing data imports also have a working knowledge of the corresponding manual data entry functionalities in the system.

The data import/export functionality also allows for linkage to existing health information systems by import or export of relevant disease case data. Initial incompatibility issues are expected, especially with regards to names of geo entities, due to inconsistency in spellings or name changes. For geo entities, this can be addressed through an import synonym tool which is included in the system and assists the user in finding names for a given geo entity that closely resembles the one the user is attempting to include in the import. The user then can add the name of the geo entity from the external health information system as a synonym for the same geo entity in the geographical entity tree in our system, which will facilitate subsequent data imports. Finally, the system still lacks capacity for mass-deletion of data, which can become an issue if the data import process is not handled carefully.

### Information Trees

The information trees (geographical entity tree, universal tree and term tree) provide tremendous adaptability in the system but require careful consideration when they are configured for a local implementation. There is no question that poorly configured information trees will lead to downstream problems with the operational use of the system. It is important that the team executing the initial configuration of the information trees has domain expertise relating to vector and disease surveillance and control practices in the local environment, as well as a clear idea of what the local user wants to get out of the system in terms of specific outputs to support decision-making and reporting.

The system comes with a default term tree and the universal tree is not onerous to configure. Data for geo entities can be imported into the system by the user to build a locally relevant geographical entity tree. This is, however, restricted to import for a single geographical hierarchical level at a time, which can make the process of building the geographical entity tree time-consuming. Alternatively, the data making up the geographical entity tree can be mass-imported but this currently requires assistance by software developers.

### Statistical and Spatial Analysis Capacity

The system was developed primarily to support operational disease control programs and therefore has very limited statistical and spatial analysis capacity. Statistical operations that are directly supported in the system are restricted to 1) query builder calculations of sums, averages, and minimum and maximum values and 2) pre-configured query builder custom calculations that relate to specific system functionalities, such as disease case incidence or mosquito abundance indices. Other statistical operations require the user to export data for subsequent import into a statistical software package.

The system supports, through the use of GeoServer/OpenLayers, basic mapping functions but essentially lacks spatial analysis capacity. The system is capable of producing map overlays to illustrate spatial patterns, for example disease case incidence in relation to percentage coverage by a given control intervention, but lacks capacity for applying spatial statistics to further explore these patterns. To achieve this, the user needs to export a shapefile from the system for subsequent import into a GIS software with spatial analysis capacity.

### Future Directions

The most important short-term future directions are pilot implementations of the system, including assessments of the cost for system set-up and operation, and the inclusion in the multi-disease data management system platform of additional important vector-borne diseases such as Chagas disease, human African trypanosomiasis, leishmaniasis, lymphatic filariasis and onchocerciasis.

Additional future plans include 1) making the system directly compatible with hand-held mobile data capturing technologies, such as Personal Digital Assistants and smartphones, 2) developing an over-arching query builder to make it easier to combine data from different parts of the system, 3) developing additional user-configurable functionalities such as configurable indicator surveys or knowledge, attitudes and practices surveys and 4) determining the potential for expanding the system to include infectious diseases with other modes of pathogen transmission than arthropod vectors (e.g., through ingestion of water or direct human-to-human contact).
